# Does activating brown fat contribute to important metabolic benefits in humans? Yes!

**DOI:** 10.1172/JCI175282

**Published:** 2023-12-01

**Authors:** Aaron M. Cypess

**Affiliations:** Diabetes, Endocrinology, and Obesity Branch, Intramural Research Program, National Institute of Diabetes and Digestive and Kidney Diseases, National Institutes of Health, Bethesda, Maryland, USA.

Adipose tissues are organs that have vital physiological roles in human health and disease. Functionally, white adipose tissue (WAT) is the principal repository for triglyceride energy, while brown adipose tissue (BAT) consumes fat and glucose to generate heat via chemical uncoupling and futile cycling (1). Excess calories stored in WAT lead to overweight and obesity and cause dysfunction in many organ systems, including WAT and BAT themselves. Reversing the ravages of obesity requires a net negative energy balance, which can be achieved through a combination of reduced food consumption, reduced caloric absorption, and increased energy expenditure. For decades, it has been recognized that chronic adrenergic stimulation of rodent BAT, either physiologically by cold exposure or pharmacologically via adrenergic receptor (AR) agonists, leads to a range of metabolic benefits, including resistance to diet-induced obesity (DIO), improved glycemia, and improved lipoprotein and cardiovascular risk profile (2). In this context, the question emerged: if adult humans had functional BAT, could its activation and growth be utilized to treat obesity and related metabolic diseases?

The first step toward answering this question was conclusively established by 2009 with the demonstration of the presence of functional BAT in adult humans (3). More recently, investigators have devoted efforts to the next step, establishing at the cellular level that mouse and human adipocytes have similar genetic and functional features, particularly related to thermogenesis and the release of soluble endocrine mediators such as peptide hormones, bioactive lipids, and extracellular vesicles (4). In parallel with this second step, clinical researchers first showed that acute stimulation of human BAT by either mild cold exposure or activation of the β3-AR with mirabegron increased BAT thermogenesis and glucose uptake (5, 6). Next, they determined that chronic stimulation increased BAT mass and metabolic activity (6). By 2023, it is well accepted that adult humans have functional BAT, that it behaves like rodent BAT at the cellular level, and that it can be stimulated acutely and chronically. In addition, it is accepted that BAT has physiological relevance in human children, where it contributes to keeping them warm given their small size and limited muscle mass (7). However, a gnawing concern was that, despite similarities among the adipocytes, at the organismal level, rodent and adult human BAT were fundamentally different. Adult humans are three orders of magnitude larger than mice and therefore have a much smaller surface area–to–volume ratio that fundamentally alters their thermal dynamics: rodents must generate heat to maintain body temperature, and BAT plays a central role at 2%–5% of body weight. In contrast, humans generally maintain body temperature using what has been termed the “waste” heat from metabolic processes, with adult BAT typically only composing 0.1% to 0.5% of body weight (1). At this time, the field is in a state of balanced opposition, which led the organizers of ENDO 2023 to propose a debate between Dr. André Carpentier and me to address the question: “does activating BAT contribute to important metabolic benefits in humans?” Three specific benefits were addressed: (a) glucose metabolism; (b) cardiometabolic disease; and (c) obesity. The answer I defended was “Yes”, as detailed below.

## Glucose metabolism

In whole-body metabolic imaging studies by Carpentier and colleagues, it was shown that per gram of tissue, cold-activated BAT has one of the highest rates of both glucose and free fatty acid (FA) uptake in the body (5). However, with an estimated BAT mass of less than 100 g in many adults, even high nutrient uptake rates translated to under 10 g/day glucose removed from the blood, compared to a tenfold higher uptake possible by skeletal muscle (8). In contrast to rodents, it appears that activation of human BAT does not typically have an impact on plasma glucose comparable to insulin administration or intense exercise. At the same time, several clinical trials raised the possibility that activated BAT could help lower blood glucose. For example, ten days of cold acclimation increased BAT activity and peripheral insulin sensitivity in people with type 2 diabetes by over 40% (9). Corroboration of the dual goals of increased BAT mass and improved glucose tolerance came from two articles published in the *JCI* in 2020. Our group treated 14 healthy women of diverse ethnicities with 100 mg/day mirabegron for four weeks. This treatment doubled detectable BAT mass, increased resting energy expenditure, and raised plasma levels of the beneficial lipoprotein biomarkers HDL and ApoA1, as well as total bile acids. In addition, an i.v. glucose tolerance test revealed higher insulin sensitivity, glucose effectiveness, and insulin secretion (6). Similar benefits in glucose metabolism were reported by Finlin et al. (10), where 13 people with obesity were treated with oral mirabegron (50 mg/day) for 12 weeks. Clinical responses included improved oral glucose tolerance, reduced hemoglobin A1c levels, and improved insulin sensitivity and pancreatic β-cell function. Thus, BAT activation in humans may have the potential to increase the BAT mass, augment energy expenditure, and improve metabolic status.

Mechanistic explanations for these beneficial effects come from a clinical-translational study showing that cold exposure of either mouse or human BAT activated the enzyme 12-lipoxygenase, which generated the 20-carbon omega-3 metabolite 12-hydroxyeicosapentaenoic acid (12-HEPE). 12-HEPE is, therefore, a BAT-derived bioactive lipid that improved glucose metabolism by promoting glucose uptake into adipocytes and skeletal muscle through activation of an insulin-like intracellular signaling pathway (11). In summary, prospective clinical trials have already shown that targeted activation of human BAT leads to the release of at least one bioactive lipid, 12-HEPE, that induces tissue glucose uptake. In parallel, chronic activation by cold exposure or the β3-AR agonist mirabegron improves glucose tolerance. Another set of clinical trials will be needed to show the proportional contribution of BAT-derived adipokines on glucose metabolism in humans.

## Cardiometabolic disease

Research on mouse FA metabolism has shown how triglyceride-rich lipoproteins (TRL) are taken up through a sequence of tissue-specific steps, starting within the BAT vasculature. Using the APOE*3-Leiden.CETP mouse model, which expresses human APOE and LDLR and develops atherosclerosis, investigators saw that BAT activation led to increased energy expenditure, decreased plasma triglyceride and cholesterol levels, and reduced atherosclerosis (12, 13). Mouse BAT is also involved in bile acid processing, the metabolism of which is connected directly to the distribution of dietary cholesterol. Cold exposure triggered postprandial TRL uptake in BAT, which accelerated cholesterol delivery to the liver. BAT activation increased synthesis of bile acids, promoting their biliary excretion, and higher bile acid levels in the gut led to an altered microbiome (14).

The possibility that activated BAT could lead to an improved cardiometabolic profile in humans is supported by a very large retrospective study: 134,529 ^18^F-FDG PET/CT scans from 52,487 patients undergoing cancer surveillance, who were categorized as BAT+ and BAT–**.** Using propensity score matching, the authors showed that the BAT+ patients had comparatively beneficial blood glucose, triglyceride, and HDL values across a range of BMI’s. These patterns were associated with lower prevalence of cardiometabolic diseases, including type 2 diabetes, dyslipidemia, coronary artery disease, cerebrovascular disease, congestive heart failure, and hypertension (15). A smaller, prospective evaluation of human BAT showed similar results: over five years, cold-induced BAT activity correlated with lower levels of cardiovascular risk factors and carotid intima-media thickness, yet higher carotid elasticity (16). A mechanistic explanation came from translational studies showing that cold exposure in mice increased the enzymatic activity of soluble epoxide hydrolases expressed only in mouse BAT and led to the production of the bioactive lipid 12,13-dihydroxy-9Z-octadecenoic acid (12,13-diHOME). Injection of 12,13-diHOME in mice acutely activated BAT fuel uptake by promoting the translocation of the FA transporters FATP1 and CD36 to the cell membrane and FA uptake into brown adipocytes. In addition to improving cold tolerance, 12,13-diHOME lowered serum triglycerides. Support for this process happening in humans came from a clinical trial showing that cold exposure increased the levels of plasma 12,13-diHOME in healthy humans (17).

Bolstering the role of BAT-derived bioactive lipids in cardiometabolic health goes beyond cold exposure. It was shown that a bout of moderate-intensity exercise increased BAT-derived circulating 12,13-diHOME levels in mice of different sexes, ages, and levels of activity. Treatment with 12,13-diHOME increased skeletal muscle FA uptake and oxidation (18). Could chronic BAT activation in humans lead to improved cardiometabolic risk and ultimately clinical endpoints? In addition to the retrospective data supporting this possibility, O’Mara et al. showed that four weeks of mirabegron administration increased serum HDL and total plasma bile acids (6). Therefore, as with improvements in glucose metabolism, chronic activation of human BAT may lead to beneficial cardiometabolic health through the release of bioactive lipids and other adipokines.

## Obesity

There are two ways that activated BAT could directly impact human obesity, through a suppression of appetite and/or an increase in energy expenditure. In mice, the latter approach can be effective, as seen with the transplantation of 100 mg of BAT into mice followed by 12 weeks of a high-fat diet (HFD) (19). However, currently the overall picture is more ambiguous. Intermittent cold exposure of mice fed a HFD increased energy expenditure and transiently improved glucose tolerance, but there was no weight loss due to a compensatory increase in food intake (20). These results reflect a general pattern in mammals: interventions that increase energy expenditure, such as cold exposure, adrenergic treatment, or exercise, over time also increase food intake. Therefore, it is more likely that these approaches may help metabolic status but will not achieve a long-term net negative energy balance. Prospective clinical trials using either cold exposure or mirabegron have consistently shown increases in detectable BAT metabolic activity and volume with no changes in appetite or weight (6, 10, 21, 22). This perspective could change should it be established that activated BAT releases peptides or metabolites associated with appetite suppression, which would then be validated through longer-duration clinical trials.

## Summary

**Figure 1:**
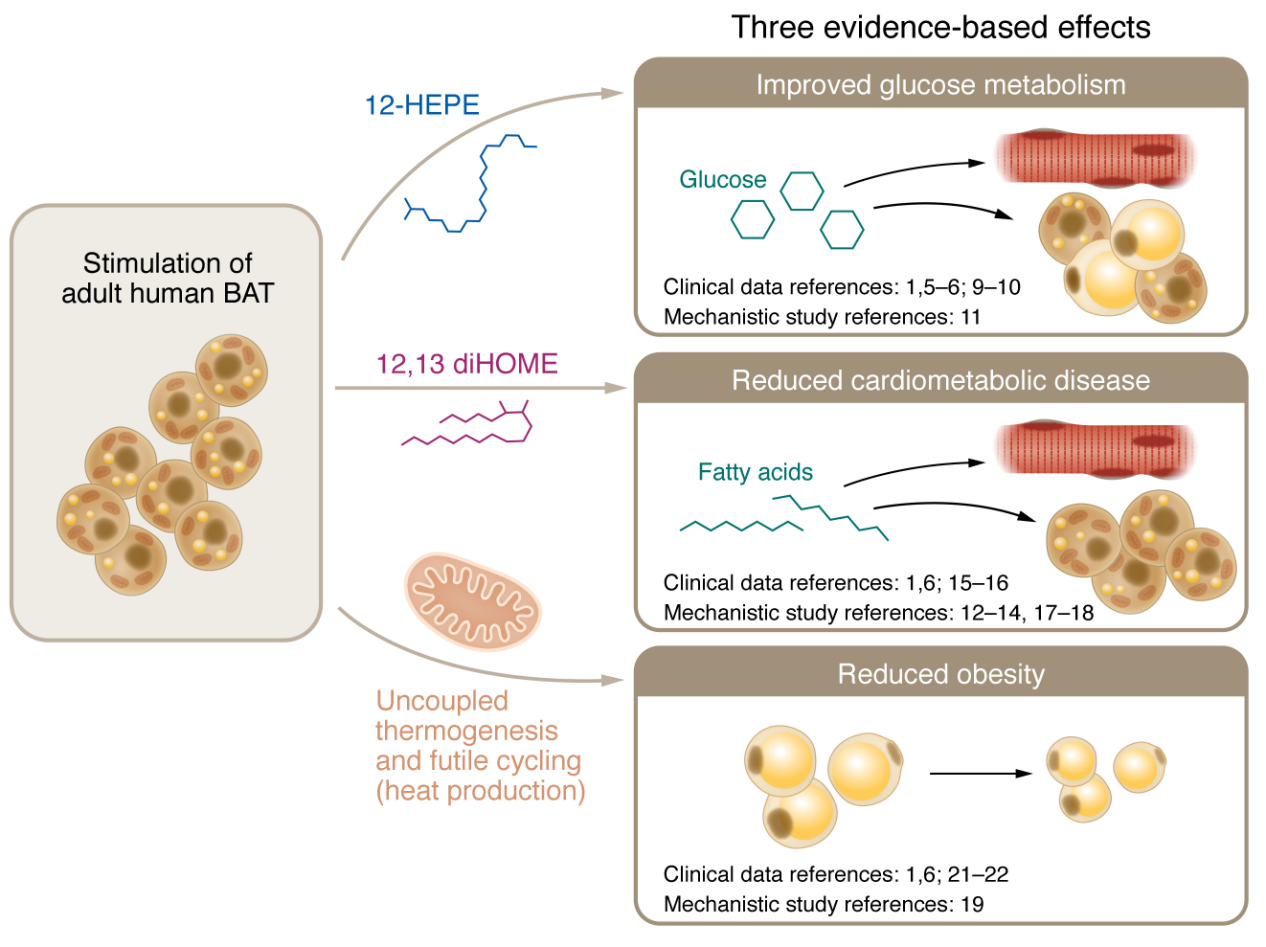
Activated BAT produces three metabolic benefits via evidence-based mechanisms. Human BAT responds to cold, pharmacological adrenergic, and other mechanisms of stimulation by consuming glucose and fatty acids to produce mediators and generate heat via chemical uncoupling and futile cycling. Evidence established via clinical trials and mechanistic studies supports improvement to metabolism with three measurable effects: improved glucose metabolism, improved cardiovascular disease, and improved obesity.

In the two decades since the discovery of functional BAT in adult humans, there has been intense interest in whether BAT activation could lead to metabolic benefit. The debate at ENDO 2023 considered the three-part question: could BAT activation be a treatment, or an adjunct treatment, for disorders associated with glucose metabolism, cardiometabolic disease, and/or obesity? The timing of the debate was auspicious because there was clinical and translational evidence; however, the data supported both sides, but no studies definitively answered these questions. A major factor undermining the physiological relevance of human BAT activation is the quantity of BAT; contemporary adult humans have insufficient BAT mass to achieve metabolic benefit by itself — even though BAT has one of the highest rates of glucose and FA consumption and energy expenditure per gram of tissue in the body. Thus, one tactic for achieving metabolic benefit might be to increase human BAT mass, either via cold exposure or pharmacologically. Further research is needed to determine whether this approach is feasible and effective. Rather, I took the perspective that BAT has both functional and endocrine roles, and the latter likely explains the mechanism by which activated human BAT can produce metabolic benefit. I asserted “yes” for each of the three aspects and provided evidence from a combination of retrospective studies, in vitro human and in vivo mouse models, and prospective clinical trials (Figure 1). The common feature in all three situations is that human activated BAT produces wide-ranging metabolic benefits through the release of secreted factors, peptides, bioactive lipids, and other mediators. The field is now in a position where the next two decades will witness the completion of the investigations necessary to determine whether activating BAT contributes to important, meaningful metabolic benefits in humans.

## Version 1 12/01/2023

Electronic publication
